# Biomimetic Total Synthesis of (±)-Lappaceolides A and B

**DOI:** 10.1021/acs.orglett.5c02445

**Published:** 2025-10-01

**Authors:** Rajanish R. Pallerla, Jenna Hakola, Leevi Härkönen, Juha H. Siitonen

**Affiliations:** Department of Chemistry and Materials Science, 174277 Aalto University, Kemistintie 1, FI-02150 Espoo, Finland

## Abstract

The first total synthesis of lappaceolides A and B is achieved in two steps by using a biomimetic vinylogous-Michael–oxa-Michael domino reaction. The domino reaction proceeds with Cs_2_CO_3_ in 1,2-DCE at elevated temperatures and requires careful kinetic control. The total synthesis provides further proof of the biosynthetic hypothesis of lappaceolides being dimers of the natural product siphonodin.

## Graphical abstract

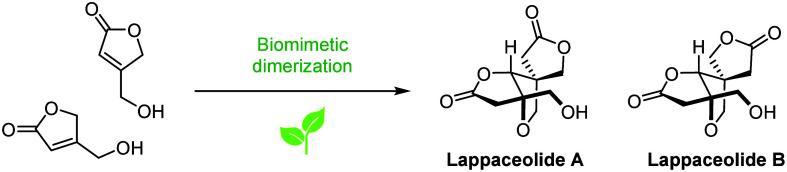

Lappaceolides A (**1**) and B (**2**) were isolated in 2005 from seeds of rambutan (*Nephelium lappaceum*) by the Ragasa group.^1^ During the isolation studies a known natural product, siphonodin (**3**), was also co-isolated from the seeds.^2^ Structurally, lappaceolides A (**1**) and B (**2**) are tricyclic monoterpene lactones that differ in their C5 spiro configuration. Lappaceolides house three contiguous stereogenic centers (C2, C5, C3), two of which are fully substituted. During the isolation studies lappaceolides (**1** and **2**) were postulated to arise from a Michael/oxa-Michael dimerization of siphonodin (**3**), but the biosynthesis has not been elaborated further since.^1^ During the isolation studies, the naturally occurring 10:7 mixture of lappaceolides A and B was found to have a nonzero optical rotation. Individual diastereomers lappaceolides A and B were separated and purified using chiral stationary phase HPLC to deliver enantiopure materials. Due to chromatographic enantioenrichment, it is not possible to evaluate the exact enantiopurities of lappaceolides A and B in Nature. We set out to verify the Ragasa biosynthetic proposal by developing a biomimetic total synthesis of racemic lappaceolides A (**1**) and B (**2**).

Devising a total synthesis approach around Ragasa’s siphonodin (**3**) homodimerization hypothesis, the C3–O bond of lappaceolides A (**1**) and B (**2**) can be disconnected in an oxa-Michael sense, yielding dimeric diol **4** (Scheme 1B). This dimeric compound **4** can be further disconnected in a vinylogous Michael addition, yielding two molecules of siphonodin (**3**). With these disconnections in hand, we envisioned that they could be combined in the forward sense into a vinylogous-Michael–oxa-Michael domino reaction to assemble both lappaceolides A (**1**) and B (**2**) directly from siphonodin (**3**) in a single step.^3^ As both lappaceolides **1** and **2** are enantioenriched, the vinylogous-Michael–oxa-Michael domino reaction was originally proposed to be enzymatic in Nature.^1^ In this proposal, the absolute configuration of **1** and **2** would be set by the initial vinylogous Michael reaction. Such enantioselective enzymatic and organocatalytic vinylogous-Michael reactions have been reported in the literature.^4a,4b^ Alternatively, lappaceolides could also be initially produced as racemates and later degraded enzymatically, leading to downstream enantioenrichment.^5^ At this point, it is impossible to differentiate between the alternative explanations.

**Scheme 1:**
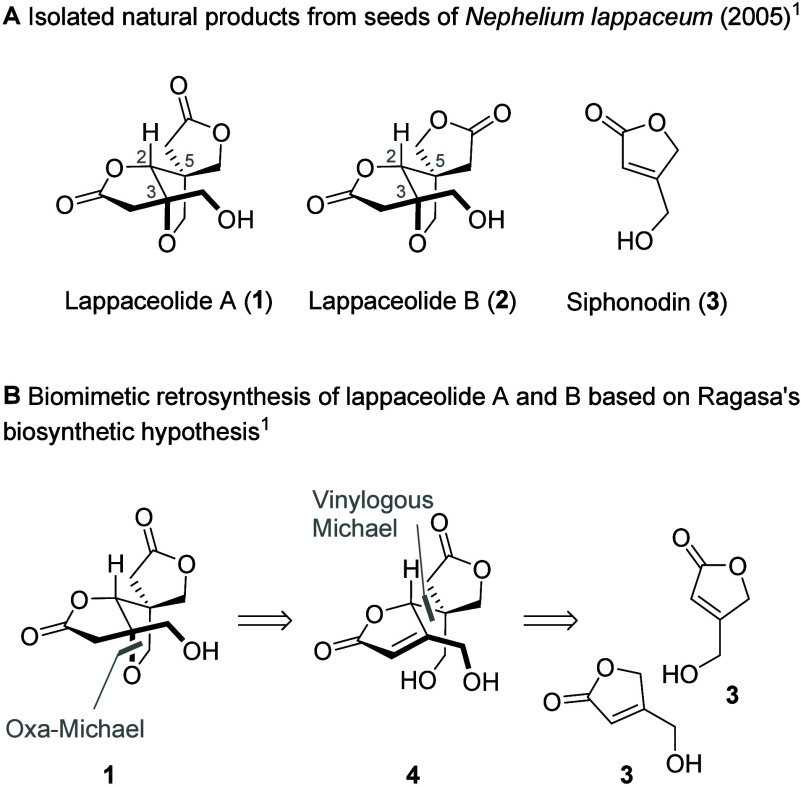
(A) Natural Products from *Nephelium lappaceum*; (B) Biomimetic Retrosynthetic Analysis of **1** and **2**

Based on the above analysis, the campaign was initiated by synthesis of the postulated biosynthetic monomer siphonodin (**3**). This was achieved by treating dihydroxyacetone (**7**) with the ylide **8**, resulting in a Wittig-olefination–lactonization domino reaction to deliver the siphonodin (**3**) in 78% yield (Scheme 2).^6a,6b^ The approach was readily scalable, allowing production of multigram quantities of siphonodin (**3**).

**Scheme 2:**
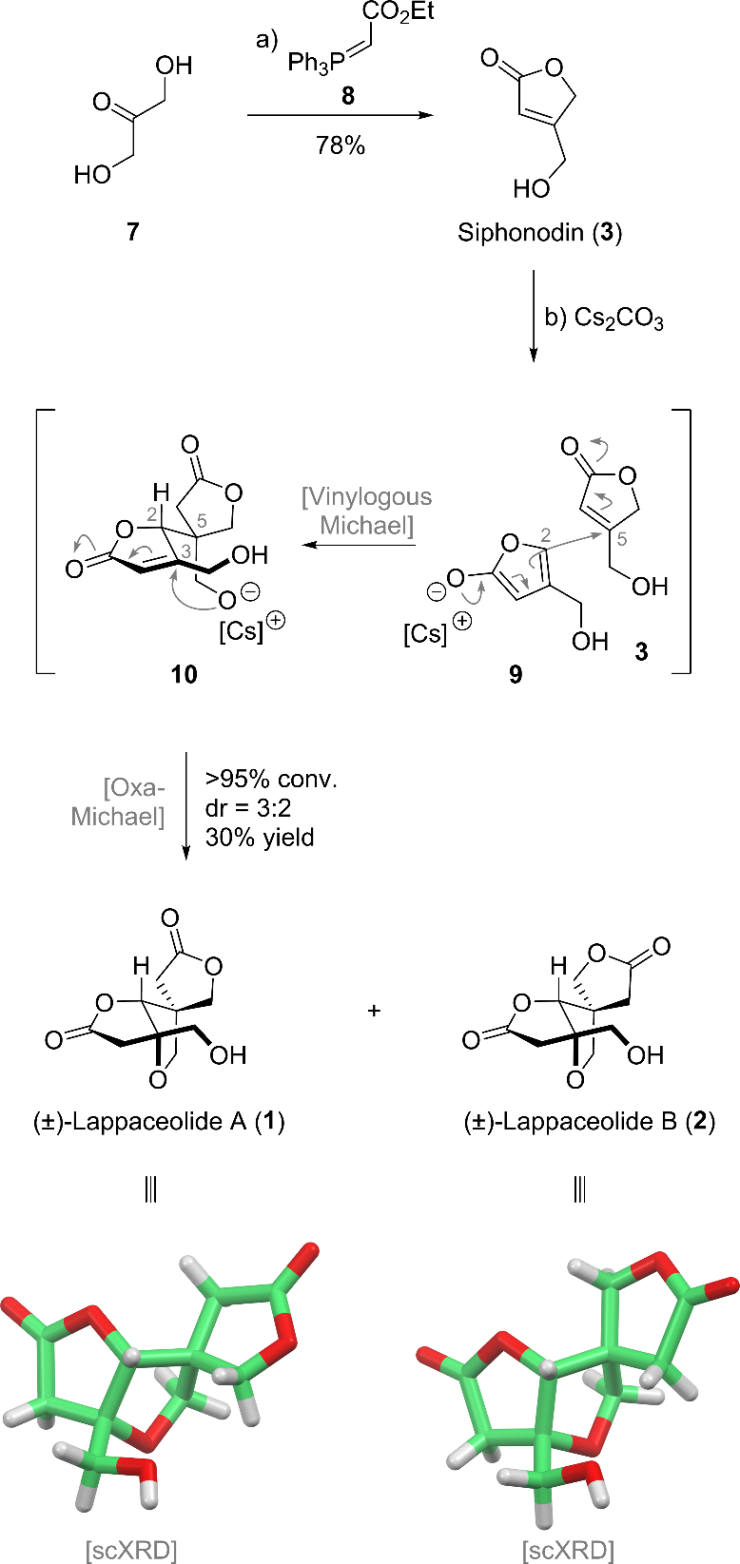
Total Synthesis of Lappaceolides A (**1**) and B (**2**)­^a^

a Conditions: a) **8** (1.1 equiv), DCM, rt, 24 h, 78%; b) Cs_2_CO_3_ (10.0 equiv), 1,2-DCE, 85 °C, 4 h, 30%.

We then turned our attention to the biomimetic vinylogous-Michael–oxa-Michael homodimerization of **3**. Despite the mechanistically promising biomimetic domino reaction, numerous attempts at dimerizing **3** were unsuccessful. Dimerizing **3** at room temperature or elevated temperatures in a range of solvents (THF, water, acetonitrile, DCM, PhMe) with a range of bases (K_2_CO_3_, Na_2_CO_3_, Cs_2_CO_3_, TBAF, Et_3_N, DBU, NaH, DABCO, NaHMDS, KO*t*Bu) and acidic additives (Zn­(OTf)_2_, Amberlyst 15, and *p*-TsOH) gave no detectable (^1^H NMR) conversion of **3** to lappaceolides (see the SI).

A related problem was faced by the Lawrence group in their total synthesis of (−)-angiopterlactone B while developing a domino reaction housing a Michael–oxa-Michael sequence.^7^ Implementing the Lawrence conditions, using 20 mol % of K_2_CO_3_ in 1,2-DCE at 70 °C, in our system, we found a 5% conversion to lappaceolides A (**1**) and B (**2**) in dr = 54:46 after 12 h. Taking this initial discovery through extensive further optimization (see the SI), we found that using 10.0 equiv of Cs_2_CO_3_ in 1,2-DCE at 85 °C in 4 h results, in the best case, in 100% conversion to lappaceolides A (**1**) and B (**2**) in a 3:2 ratio and 30% isolated yield. The yields, conversions, and diastereomeric ratios of **1** and **2** were however found to vary (8–30%, 75–100%, dr 3:2–2:3) from batch to batch. Reaction times longer than 4 h were found to result in lower yields, indicating that **1** and **2** are unstable at elevated temperatures under the reaction conditions. Careful kinetic control was, therefore, required for a successful dimerization of **3**.

Purification of lappaceolides A (**1**) and B (**2**) proved to be equally challenging. Using typical flash column chromatography eluent systems such as EtOAc/hexane or DCM/MeOH did not allow unreacted siphonodin (**3**) to be separated from **1** and **2**. Ultimately, flash column chromatography using Et_2_O–MeCN–DCM (1:1:3 v/v/v) allowed the separation of siphonodin (**3**) from lappaceolides (**1** and **2**). The spectroscopic data obtained for the synthetic sample of lappaceolides A (**1**) and B (**2**) were identical to those reported for the isolated materials.^1^ Furthermore, a single-crystal X-ray diffraction structure of cocrystallized lappaceolides A (**1**) and B (**2**) was obtained.^8^

The domino reaction **3** → **1** and **2** proceeds through a vinylogous Michael reaction between vinylogous enolate **9** and **3** followed by an intramolecular oxa-Michael reaction of the thus formed intermediate **10** (Scheme 2). The vinylogous Michael reaction sets the C2 and C5 stereogenic centers, and the facial selectivity at this stage ultimately determines the diastereomeric ratio of **1** and **2**. As the diastereoselectivity was found to vary greatly, even changing the major diastereomer from batch to batch, the step is not stereoselective. The stereogenic center at C3, however, is set in high fidelity by the intramolecular oxa-Michael reaction taking place from the bottom face of the C3 center to establish the *cis*-fused dioxabicyclo[3.3.0]­octane ring.^9^

Both lappaceolide A (**1**) and lappaceolide B (**2**) are isolated from *Nephelium lappaceum* as a 10:7 mixture, and both individual diastereomers are enantioenriched. Given the successful biomimetic dimerization of siphonodin (**3**), this observation has two explanations. Either 1) the biosynthetic enzymatic vinylogous Michael reaction **9** is counterintuitively not diastereoselective yet highly enantioselective or 2) lappaceolides are initially produced nonenzymatically as racemates and then late-stage enzymatically enantioenriched.

In conclusion, we have verified Rasaga’s biosynthetic proposal by establishing a biomimetic two-step total synthesis of lappaceolides A (**1**) and B (**2**) from siphonodin (**3**) featuring a Cs_2_CO_3_-mediated vinylogous-Michael–oxa-Michael domino reaction. The dimerization reaction is highly sensitive to reaction conditions and requires careful kinetic control to achieve high conversions, highlighting the synthetic challenges associated with developing nonenzymatic approaches to biomimetic total syntheses. We believe the biosynthesis of lappaceolides proceeds in Nature under a similar reaction mechanism.^10^

## Supplementary Material



## Data Availability

The data underlying this study are available in the published article and its Supporting Information.

## Notes

Experimental procedures, characterization data, crystallographic details, and NMR spectra (PDF)
