# Supplementary material for: Biomimetic Total Synthesis of (±)-Lappaceolides A and B
Source: Org Lett. 2025 Oct 1;27(40):11149–51. doi: 10.1021/acs.orglett.5c02445 (PMC12519463; doi:10.1021/acs.orglett.5c02445)

Supporting Information

Biomimetic Total Synthesis of (±)-Lappaceolides A and B

Rajanish R. Pallerla, Jenna Hakola, Leevi Härkönen, Juha H. Siitonen*

Department of Chemistry and Materials Science, Aalto University

Kemistintie 1, FI-02150 Espoo, Finland.

J. H. S. juha.siitonen@aalto.fi

Contents

1 General Procedures 3

2 Total Synthesis of Lappaceolides A and B 4

2.1 Siphonodin (**3**) 4

2.2 Lappaceolides A and B (**1** and **2**) 4

3 Comparison to the Isolated Material 5

3.1 NMR Comparison of Lappaceolide A (**1**) to Isolated Material 5

3.2 NMR Comparison of Lappaceolide B (**2**) to Isolated Material 6

3.3 X-ray Data for Lappaceolides A and B 7

4 Optimization of the Dimerization 11

5 References 12

6 Spectral Data 13

1 General Procedures

All reactions were carried out in non-dried glassware open to air and at room temperature (22 °C) unless otherwise noted. Solvents and reagents were used as obtained from the supplier unless otherwise noted. Analytical TLC was performed using Merck silica gel 60G F_{254} plates and analyzed by UV light or by staining upon heating with potassium permanganate solution (*1.0 g $KMnO_4$, 2.0 g Na_2CO_3, 100 mL DI H_2O*) or vanillin (*6.0 g vanillin, 100 mL EtOH, 1.0 mL conc. H_2SO_4*) or PMA (*4.8 g phosphomolybdic acid, 100 mL EtOH*). For silica gel chromatography, the Teledyne ISCO Combiflash NextGen 300+ with RediSep Gold columns or the flash chromatography technique with Merck silica gel 60 (230−400 mesh) and p.a. grade solvents were used. For preparative TLC chromatography purifications, 20×20 cm Merck silica gel 60G F_{254} plates were used. The NMR spectra were recorded on a Bruker Avance NEO 400 spectrometer. The solvent used for NMR spectroscopy were $(CD_3)_2SO$ and $CDCl_3$, using tetramethylsilane (TMS) as the internal reference. The chemical shifts are reported in ppm relative to reference TMS (δ 0.00) or residual $CHCl_3$ (δ 7.26) and $(CD_2H)(CD_3)SO$ (δ 2.50) for ^1H NMR and $CDCl_3$ (δ 77.16) and $(CD_3)_2SO$ (δ 39.52) for ^{13}C NMR. The diastereomeric ratios were determined by ^1H NMR analysis of crude reaction mixtures. All spectra were recorded at ambient temperature (298 K). Coupling constants (*J*) were reported in Hz, and chemical shift (δ) were reported in ppm. The multiplicity of signals is indicated using the following abbreviations: s = singlet, br s = broad singlet, d = doublet, t = triplet, q = quartet, quint = quintet, dd = doublet of doublets, m = multiplet. High-resolution mass spectrometric data were measured using an Agilent 6350 QTOF Mass spectrometer working in positive mode. FTIR data was acquired using a Bruker Alpha with an Eco-ATR attachment.

2 Total Synthesis of Lappaceolides A and B

2.1 Siphonodin (3)

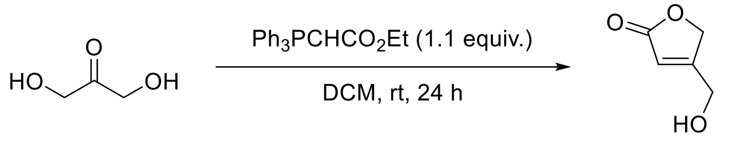

To a stirred solution of dihydroxyacetone (1.50 g, 16.7 mmol, 1.00 equiv.) in DCM (16 ml), ethyl (triphenylphosphoranylidene)acetate (6.40 g, 18.3 mmol, 1.10 equiv.) was added in four portions. The flask was loosely capped, and the resulting suspension was stirred at rt for 24 h. The reaction mixture was extracted with water (4 × 10 ml, Note 1). The combined aqueous layers were washed with DCM (3 × 5 ml), concentrated *in vacuo* and dried azeotropically with toluene (4 × 5 ml, 10 mbar, 60 °C). The material was taken up in EtOAc (40 ml) and filtered through a pad of silica. The pad was washed with EtOAc (3 × 40 ml), and the filtrate was concentrated *in vacuo* to yield siphonodin as a clear oil which crystallizes in the freezer (1.5 g, 78%). The thus obtained material was sufficiently pure for the following steps.

Note 1: Siphonodin is highly soluble in water and dissolves in the aqueous layer.

NMR data matched those reported previously.[1]

***R*f** (EtOAc): 0.37 (Vanillin stain, blue).

^{1}H NMR (400 MHz, $(CD_3)_2SO$) δ: 5.96 (quint, *J* = 1.9 Hz, 1H), 5.42 (t, *J* = 5.5 Hz, 1H), 4.87 (d, *J* = 1.9 Hz, 2H), 4.37 (dd, *J* = 5.5, 1.9 Hz, 2H).

{^{1}H}^{13}C NMR (100 MHz, $(CD_3)_2SO$) δ: 173.5 (2C), 112.7, 71.2, 57.6.

2.2 Lappaceolides A and B (1 and 2)

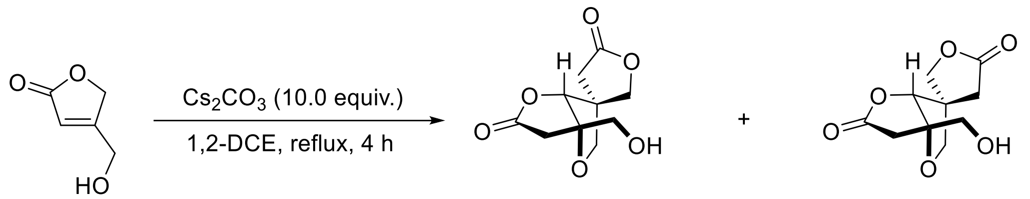

Lappaceolide A (**1**) Lappaceolide B (**2**)

A suspension of siphonodin (0.10 g, 0.88 mmol, 1.00 equiv.) and Cs_2CO_3 (2.86 g, 8.76 mmol, 10.0 equiv.) in 1,2-DCE (2 ml) was heated to reflux using an oil bath. After 4 hours the mixture was cooled to room temperature and filtered through a sintered funnel. The thus-obtained liquid was concentrated *in vacuo* and purified by silica gel column chromatography (1:1:3 MeCN/DEE/DCM) to yield an inseparable mixture of lappaceolides A and B as a white solid (30 mg, 30%, dr = 3:2).

***R*f** (1:1:3 MeCN/DEE/DCM): 0.3 (PMA stain, green).

^{1}H NMR (400 MHz, $CDCl_3$) δ: 4.79 (br s, 1H), 4.78 (br s, 0.6H), 4.63 (d, *J* = 10.4 Hz, 0.6H), 4.34 (dd, *J* = 9.7, 1.3 Hz, 1H), 4.26 (d, *J* = 9.6 Hz, 1H), 4.15 (d, *J* = 10.4 Hz, 0.6H), 4.12 (d, *J* = 9.6 Hz, 1H), 4.05 (d, *J* = 9.6 Hz,

0.6H), 3.88 – 3.82 (m, 2H), 3.81 – 3.78 (m, 1H), 3.74 – 3.69 (m, 1.6 H), 3.02 (d, *J* = 18.4 Hz, 1H), 2.82 (d, *J* = 19.2 Hz, 0.6H), 2.80 – 2.66 (m, 3.2H), 2.61 (d, *J* = 17.9 Hz, 0.6H), 2.42 (d, *J* = 18.4 Hz, 1H).

{^{1}H}^{13}C NMR (100 MHz, $CDCl_3$) δ: 174.6, 174.4, 173.7, 173.6, 89.4, 89.2, 88.8, 87.8, 74.8 (2C), 74.2, 69.2, 64.6, 64.4, 51.7, 51.0, 38.4, 37.2, 37.1, 31.8.

FTIR (ATR, cm^{-1}): 2984, 1736, 1372, 1233, 1042, 937, 845, 633, 607.

HRMS (ESI) m/z: [M-H$^-$] calcd. for $C_{10}H_{11}O_6^-$ 227.0561; found 227.0563, Δ = 0.9 ppm.

3 Comparison to the Isolated Material

3.1 NMR Comparison of Lappaceolide A (1) to Isolated Material

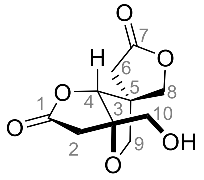

Lappaceolide A (**1**)

Table S2. Comparison of NMR data for isolated and synthetic lappaceolide A (**1**) in $CDCl_3$.[2]

Atom No.	Isolated		Synthetic		Δ^{13}C (ppm)
	^{1}H	^{13}C	^{1}H	^{13}C	
1	–	173.3	–	173.6	0.3
2	2.70 (d, *J* = 19.2 Hz, 1H)	37.0	2.70 (d, *J* = 19.3 Hz, 1H)	37.2	0.2
	2.79 (d, *J* = 19.2 Hz, 1H)		2.77 (d, *J* = 19.3 Hz, 1H)		
3	–	89.0	–	89.2	0.2
4	4.78 (br s, 1H)	87.7	4.79 (br s, 1H)	87.8	0.1
5	–	51.6	–	51.7	0.1
6	2.42 (d, *J* = 18.4 Hz, 1H)	31.7	2.42 (d, *J* = 18.4 Hz, 1H)	31.8	0.1
	3.02 (d, *J* = 18.4 Hz, 1H)		3.02 (d, *J* = 18.4 Hz, 1H)		
7	–	174.2	–	174.4	0.2
8	4,26 (d, *J* = 9.6 Hz, 1H)	74.6	4,26 (d, *J* = 9.8 Hz, 1H)	74.8	0.2
	4.34 (dd, *J* = 9.6 Hz, 1.2 Hz, 1H)		4.34 (dd, *J* = 9.7 Hz, 1.3 Hz, 1H)		
9	3.80 (dd, *J* = 9.6 Hz, 1.2 Hz, 1H)	74.1	3.81 – 3.78 (m, 1H)	74.2	0.1
	4.12 (d, *J* = 9.6 Hz, 1H)		4.12 (d, *J* = 9.6 Hz, 1H)		
10	3.72 (d, *J* = 11.6 Hz, 1H)	64.5	3.74 – 3.69 (m, 1H)	64.6	0.1
	3.86 (d, *J* = 11.6 Hz, 1H)		3.88 – 3.82 (m, 1H)		

3.2 NMR Comparison of Lappaceolide B (2) to Isolated Material

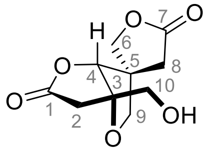

Lappaceolide B (**2**)

Table S3. Comparison of NMR data for isolated and synthetic lappaceolide B (**2**) in $CDCl_3$.[2]

Atom No.	Isolated ^1H	Isolated ^{13}C	Synthetic ^1H	Synthetic ^{13}C	$\Delta^{13}C$ (ppm)
1	–	173.4	–	173.7	0.3
2	2.68 (d, *J* = 19.2 Hz, 1H)	36.9	2.68 (d, *J* = 19.2 Hz, 1H)	37.1	0.2
	2.82 (d, *J* = 19.2 Hz, 1H)		2.82 (d, *J* = 19.2 Hz, 1H)		
3	–	89.2	–	89.4	0.2
4	4.78 (br s, 1H)	88.6	4.78 (br s, 1H)	88.8	0.2
5	–	50.9	–	51.0	0.1
6	2.61 (d, *J* = 18.0 Hz, 1H)	38.3	2.61 (d, *J* = 17.9 Hz, 1H)	38.4	0.1
	2.75 (dd, *J* = 18.0 Hz, 1.2 Hz, 1H)		2.80 – 2.66 (m, 1H)		
7	–	174.4	–	174.6	0.2
8	4.15 (d, *J* = 10.4 Hz, 1H)	69.0	4.15 (d, *J* = 10.4 Hz, 1H)	69.2	0.2
	4.63 (d, *J* = 10.4 Hz, 1H)		4.63 (d, *J* = 10.4 Hz, 1H)		
9	3.85 (dd, *J* = 9.6 Hz, 1.2 Hz, 1H)	74.6	3.88 – 3.82 (m, 1H)	74.8	0.2
	4.05 (d, *J* = 9.6 Hz, 1H)		4.05 (d, *J* = 9.6 Hz, 1H)		
10	3.73 (d, *J* = 11.6 Hz, 1H)	64.3	3.74 – 3.69 (m, 1H)	64.4	0.1
	3.83 (d, *J* = 11.6 Hz, 1H)		3.88 – 3.82 (m, 1H)		

3.3 X-ray Data for Lappaceolides A and B

Single-crystal X-ray diffraction analyses were performed on Rigaku XtaLAB Synergy R with CuKα (λ = 1.54184 Å) radiation. Crystals were obtained by slow evaporation of mixture of **1** and **2** in ethyl acetate, then suspended in protective oil and were mounted on MiTeGen loop for measurement. The data reduction and absorption corrections were made by program CrysAlisPro[3]. The structures were solved by using SHELXT[4] in OleX 2-1.5[5] and refined with SHELXL[6]. All non-hydrogen atoms were refined anisotropicaly. The hydrogen atoms were calculated to their idealised positions as riding atoms with isotropic thermal parameters as 1.2 × C for C(H) and C(H,H) and 1.5 × O for the alcohol O(H). Details about applied restrains and constraints can be found in the CIF file. The structures were drawn with Mercury[7].

**Identification code**	**Lappaceolide_A_B**
CCDC	2387316
Empirical formula	$C_{10}H_{12}O_6$
Formula weight	228.20
Temperature/K	121.4(2)
Crystal system	monoclinic
Space group	$P2_1/c$
a/Å	14.2884(3)
b/Å	10.8391(2)
c/Å	6.34558(12)
α/°	90
β/°	98.7206(19)
γ/°	90
Volume/Å^3	971.40(3)
Z	4
ρ_{calc}g/cm^3	1.560
μ/mm^{-1}	1.125
F(000)	480.0
Crystal size/mm^3	0.091 × 0.077 × 0.036
Radiation	Cu Kα (λ = 1.54184)
2Θ range for data collection/°	6.258 to 158.17
Index ranges	$-18 \le h \le 18, -13 \le k \le 13, -8 \le l \le 7$
Reflections collected	16504
Independent reflections	2075 [R_{int} = 0.0361, R_{sigma} = 0.0238]
Data/restraints/parameters	2075/22/155
Goodness-of-fit on F^2	1.051
Final R indexes [I>=2σ (I)]	R_1 = 0.0485, wR_2 = 0.1288
Final R indexes [all data]	R_1 = 0.0523, wR_2 = 0.1319
Largest diff. peak/hole / e Å^{-3}	0.42/–0.40

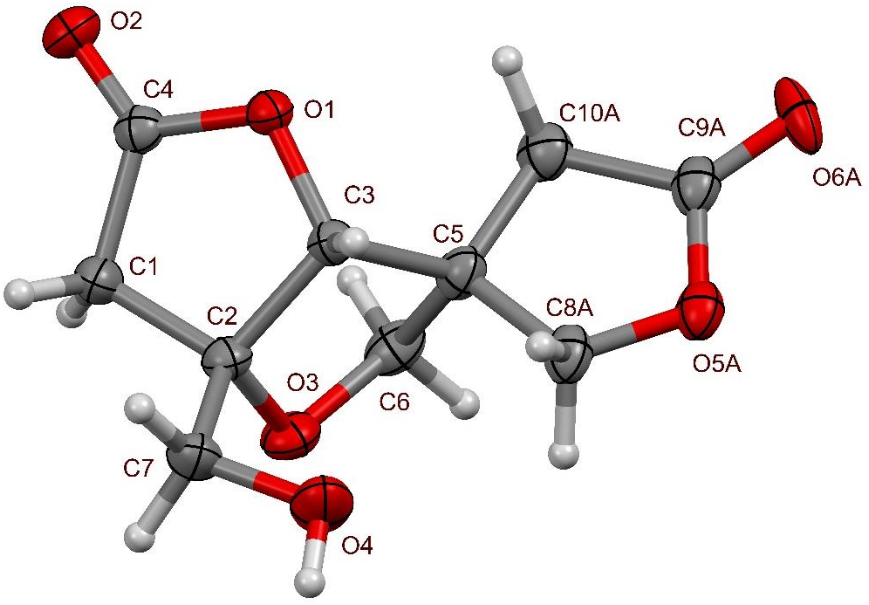

Figure S1. The thermal ellipsoid plot of **lappaceolide A.** The thermal displacement parameters are shown at 50% probability level.

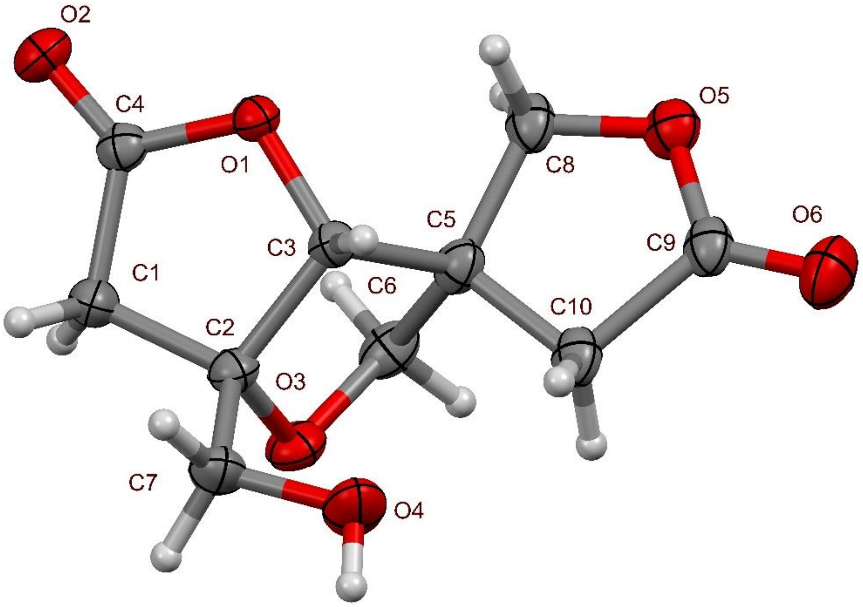

Figure S2. The thermal ellipsoid plot of **lappaceolide B.** The thermal displacement parameters are shown at 50% probability level.

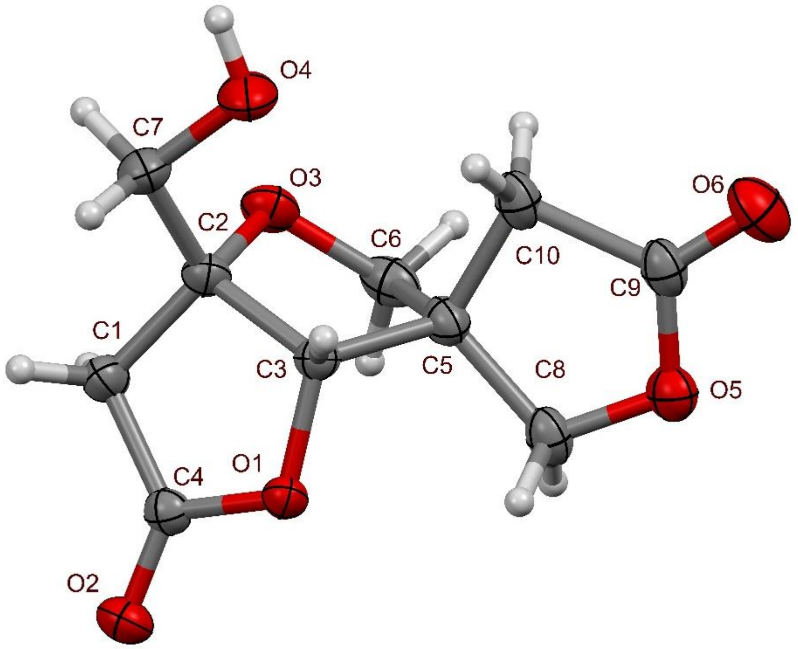

Figure S3. The thermal ellipsoid plot of **lappaceolide B enantiomer.** The thermal displacement parameters are shown at 50% probability level.

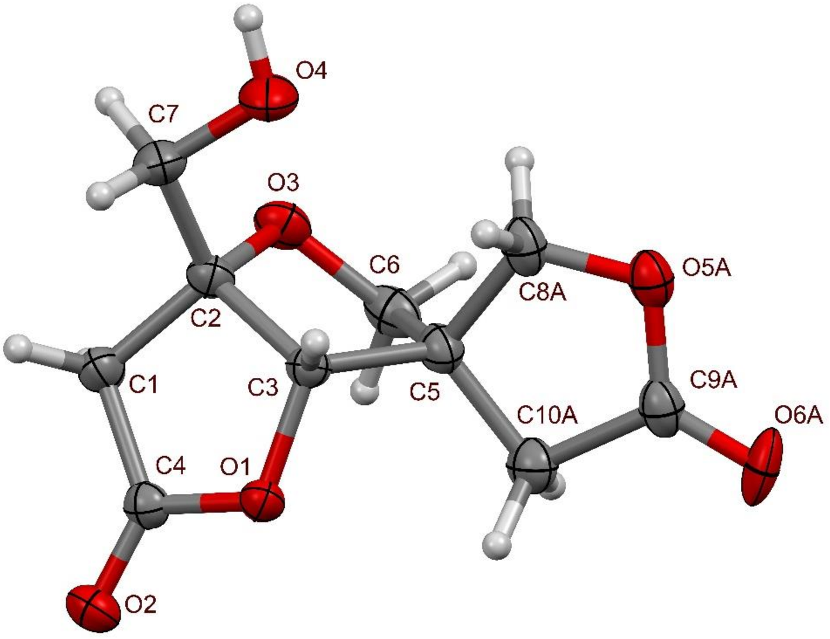

Figure S4. The thermal ellipsoid plot of **lappaceolide A enantiomer.** The thermal displacement parameters are shown at 50% probability level.

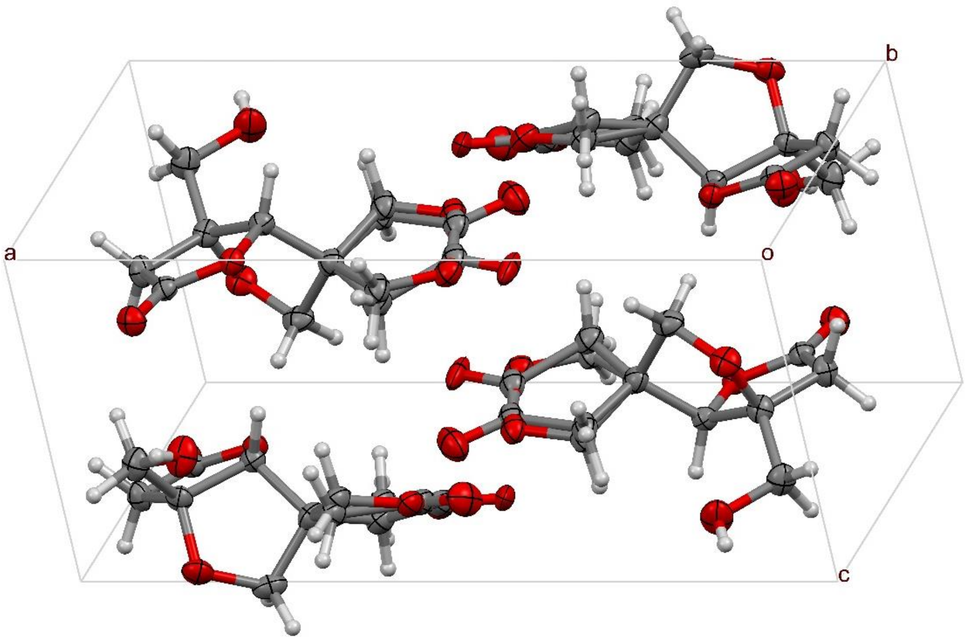

Figure S5. The unit cell packing representing the co-crystallised **lappaceolide A and B, including their enantiomers** along the *b*-axis. The thermal displacement parameters are shown at 50% probability level.

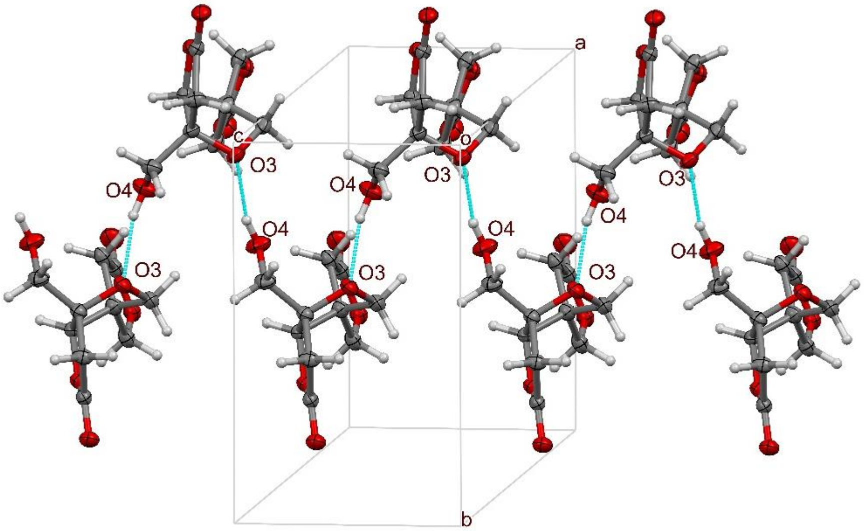

Figure S6. The crystal packing representing hydrogen bonding interaction between **O(4)**-**H** and **O(3)** of **lappaceolide B** along the *a*-axis. The thermal displacement parameters are shown at 50% probability level. Hydrogen bonds are marked with turquoise dashed lines.

4 Optimization of the Dimerization

Siphonodin (**3**, 1.0 equiv.) and base were dissolved or suspended in each solvent and either stirred at room temperature or heated. The reaction mixture was concentrated and analyzed using ^{1}H NMR.

Table S1: Reaction employed for reaction condition optimization.

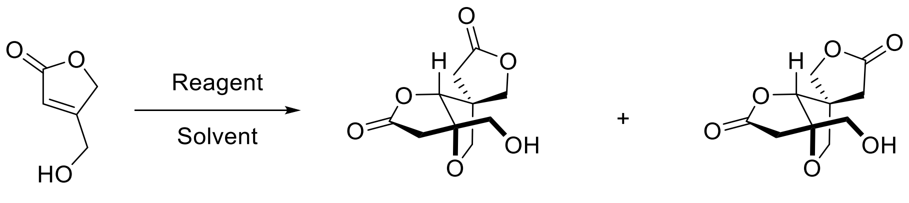

Siphonodin (**3**) Lappaceolide A (**1**) Lappaceolide B (**2**)

	Reagent (Equiv.)	Solvent	Temp (°C)	Time	Result
01	NaHMDS (1.0)	THF	–15 to 0	24 h	Decomposed
02	NaHMDS (1.0)	THF	rt	0.5 h	Decomposed
03	DABCO (1.0)	Acetone	reflux	24 h	Decomposed
04	DBU (10.0)	DCM	rt	24 h	No rxn
05	DBU (10.0) & LiCl (10.0)	MeCN	reflux	24 h	Decomposed
06	Et_3N (10.0)	$CHCl_3$	rt	24 h	No rxn
07	NaH (1.0)	THF	rt	24 h	No rxn
08	Na_2CO_3 (1.2)	Water/THF (1:1)	rt	24 h	No rxn
09	Amberlyst-15 (1.0)	DCM	rt	24 h	No rxn
10	TFA (10.0)	DCM	rt	24 h	No rxn
11	Imidazole (0.5)	DCM	rt	24 h	No rxn
12	TBAF (1.5)	THF	0 to rt	24 h	No rxn
13	CsF (1.5)	THF	0 to rt	24 h	No rxn
14	CsF (1.5)	Water/THF (1:1)	rt	24 h	No rxn
15	CsF (5.0)	Water/THF (1:1)	40	48 h	No rxn
16	KO^tBu (0.5)	THF	rt	24 h	No rxn
17	NaO^tBu (0.5)	THF	rt	24 h	No rxn
18	$Zn(OTf)_2$ (1.0)	DCM	rt	21 h	No rxn
19	$Cu(OTf)_2$ (1.0)	DCM	rt	21 h	No rxn
20	$BF_3{\cdot}OEt_2$ (1.0)	DEE	0 to rt	5 h	Decomposed
21	$TiCl_4$ (0.5)	DCM	0 to rt	5 h	Decomposed
22	$CeCl_3{\cdot}7H_2O$ (0.5)	MeOH	rt	24 h	No rxn
23	(*S*)-(+)-CSA (5.0)	MeOH	rt	24 h	No rxn
24	(*R*)-(–)-CSA (5.0)	MeOH	rt	24 h	No rxn
25	TSA (1.0)	PhMe	rt	24 h	No rxn
26	L-Proline (0.2) & Benzoic acid (0.2)	PhMe	0 to rt	24 h	No rxn
27	L-Proline (1.0)	THF	rt	24 h	No rxn
28	L-Proline (0.2) & 3-nitrobenzoic acid (0.2)	PhMe	0 to rt	24 h	No rxn

29	L-Proline (1.0)	THF	rt	24 h	No rxn
30	K_2CO_3 (1.2)	EtOAc	70	12 h	^{1}H NMR traces
31	K_2CO_3 (1.2)	1,2-DCE	85	24 h	17% conversion
32	K_2CO_3 (1.2)	1,4-dioxane	125	3 h	No rxn (microwave)
33	K_2CO_3 (5.0)	MeCN	Reflux	5 h	No rxn
34	K_2CO_3 (5.0)	Water	rt	24 h	No rxn
35	K_2CO_3 (5.0)	1,2-DCE	85	4 h	45 % conversion
36	K_2CO_3 (5.0)	1,2-DCE	85	24 h	Decomposed
37	K_2CO_3 (10.0)	1,2-DCE	85	4 h	75% conversion
38	K_2CO_3 (10.0)	1,2-DCE	85	6 h	Decomposed
39	K_2CO_3 (10.0)	1,2-DCE	85	5 h	50% conversion
40	Cs_2CO_3 (10.0)	1,2-DCE	85	4 h	80–100% conversion
41	Cs_2CO_3 (5.0)	1,2-DCE	85	4 h	50–60% conversion
42	Cs_2CO_3 (5.0)	DCM	Reflux	5 h	No rxn
43	Cs_2CO_3 (5.0)	$CHCl_3$	Reflux	5 h	No rxn
44	Cs_2CO_3 (5.0)	CCl_4	Reflux	5 h	No rxn
45	Cs_2CO_3 (5.0)	PhMe	Reflux	5 h	No rxn

5 References

1. Adam, J. M.; Foricher, J.; Hanlon, S.; Lohri, B.; Moine, G.; Schmid, R.; Stahr, H.; Weber, M.; Wirz, B.; Zutter, U. Development of a Scalable Synthesis of (*S*)-3-Fluoromethyl-γ-Butyrolactone, Building Block for Carmegliptin's Lactam Moiety. *Org. Process Res. Dev*. **2011**, *15*, 515–526.
2. Ragasa, C. Y; De Luna, R. D.; Cruz, W. C.; Rideout, J. A. Monoterpene Lactones from the Seeds of Nephelium Lappaceum. *J. Nat. Prod.* **2005**, *68*, 1394–1396.
3. CrysAlisPro 1.171.42.80a, **2023**, Rigaku Oxford Diffraction.
4. Sheldrick, G.M. *Acta Cryst*. **2015**, A71, 3-8.
5. Dolomanov, O.V., Bourhis, L.J., Gildea, R.J, Howard, J.A.K. & Puschmann, H. *J. Appl. Cryst*. **2009**, 42, 339-341.
6. Sheldrick, G.M. *Acta Cryst*. **2015**, C71, 3-8
7. Mercury 2022.3.0 (Build 392256).

6 Spectral Data

Siphonodin (**3**)
^{1}H NMR, 400 MHz
$(CD_3)_2SO$

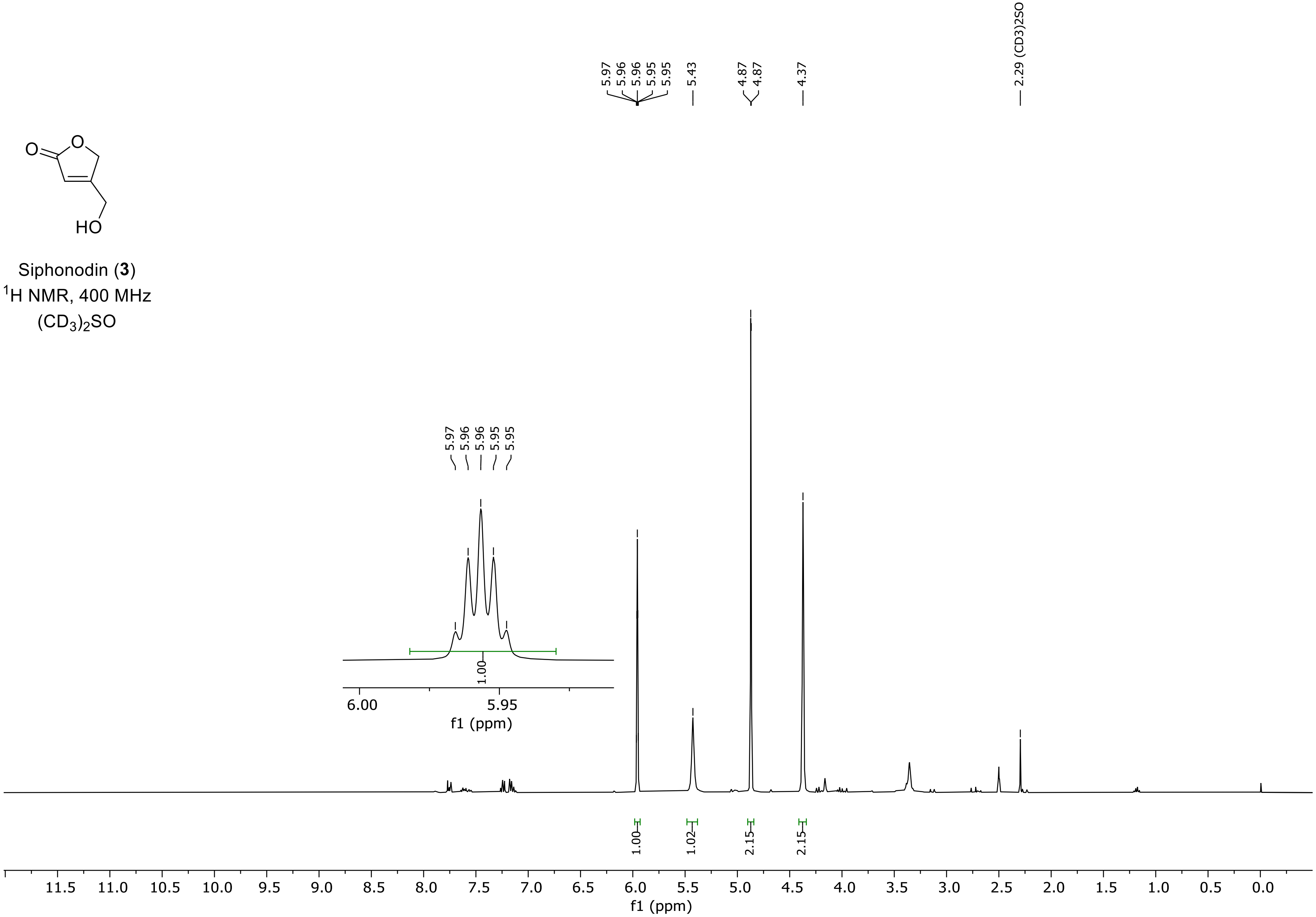

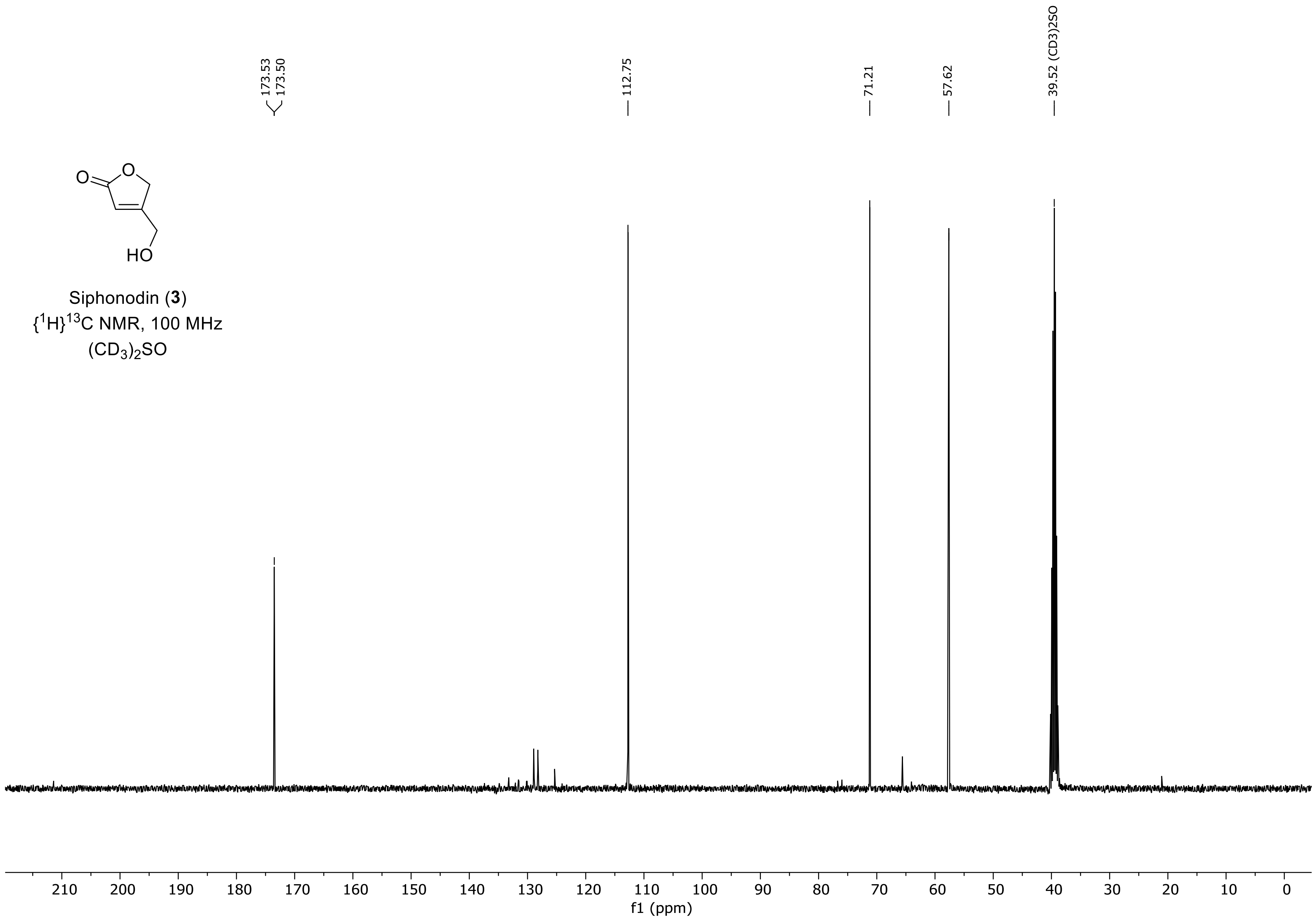
173.53
173.50
112.75
71.21
57.62
39.52 (CD3)2SO
O
O
HO
Siphonodin (3)
{1H}13C NMR, 100 MHz
(CD3)2SO
210
200
190
180
170
160
150
140
130
120
110
100
90
80
70
60
50
40
30
20
10
0
f1 (ppm)

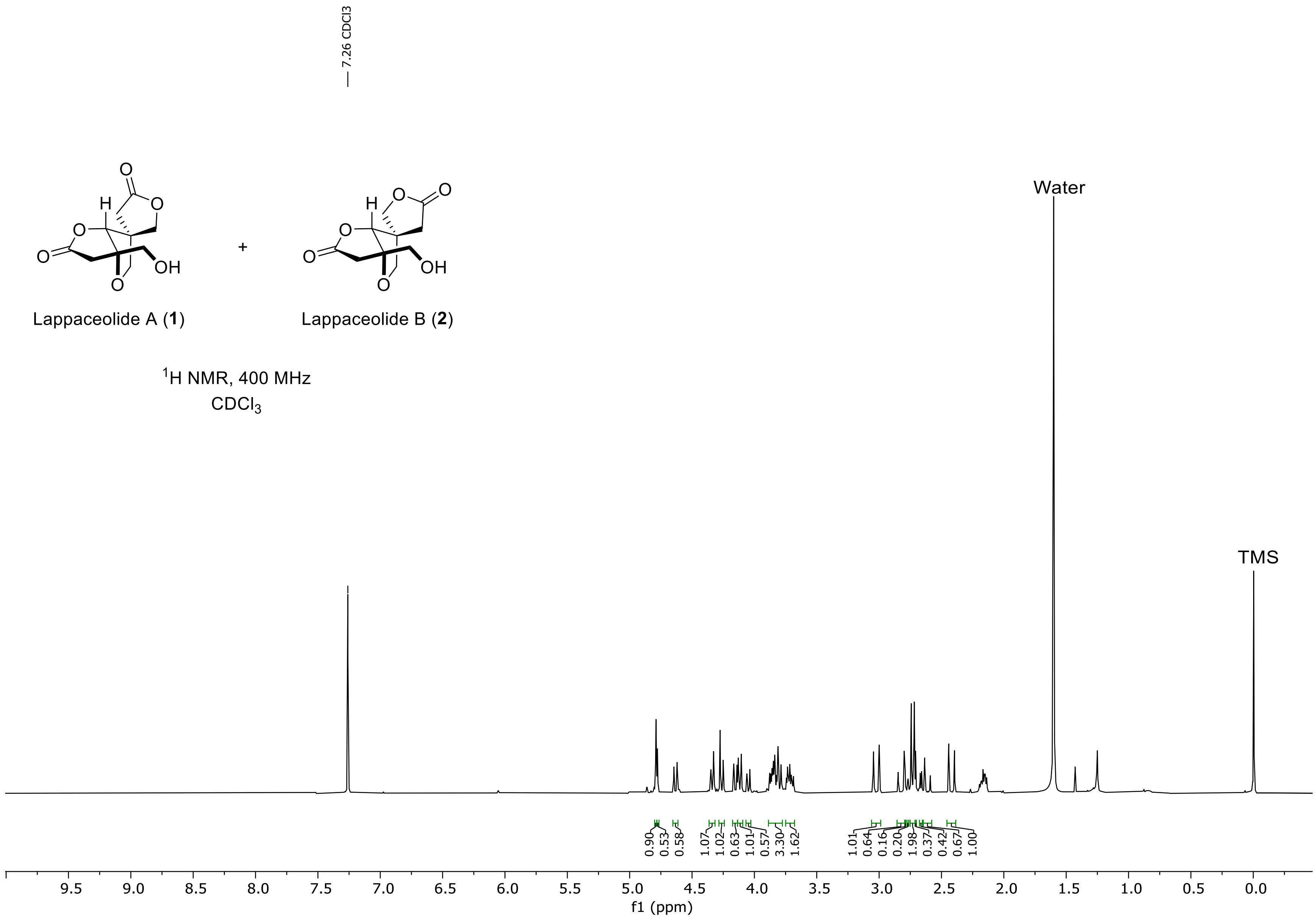
O
H
O
O
O
OH
O
Lappaceolide A (**1**)
+
H
O
O
O
O
OH
O
Lappaceolide B (**2**)
^{1}H NMR, 400 MHz
$CDCl_3$
7.26 CDCl3
Water
TMS
0.90
0.53
0.58
1.07
1.02
0.63
1.01
0.57
3.30
1.62
1.01
0.64
0.16
0.20
1.98
0.37
0.42
0.67
1.00
9.5
9.0
8.5
8.0
7.5
7.0
6.5
6.0
5.5
5.0
4.5
4.0
3.5
3.0
2.5
2.0
1.5
1.0
0.5
0.0
f1 (ppm)

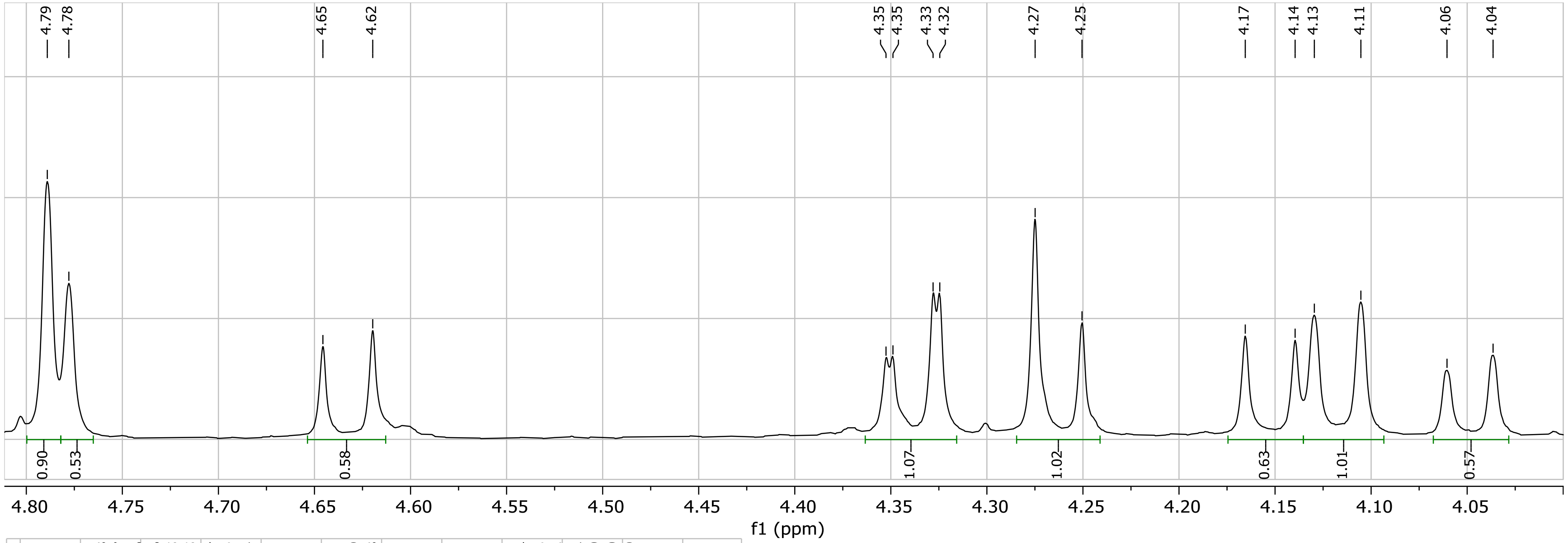
4.79 4.78 4.65 4.62 4.35 4.35 4.33 4.32 4.27 4.25 4.17 4.14 4.13 4.11 4.06 4.04
0.90 0.53 0.58 1.07 1.02 0.63 1.01 0.57
4.80 4.75 4.70 4.65 4.60 4.55 4.50 4.45 4.40 4.35 4.30 4.25 4.20 4.15 4.10 4.05
f1 (ppm)

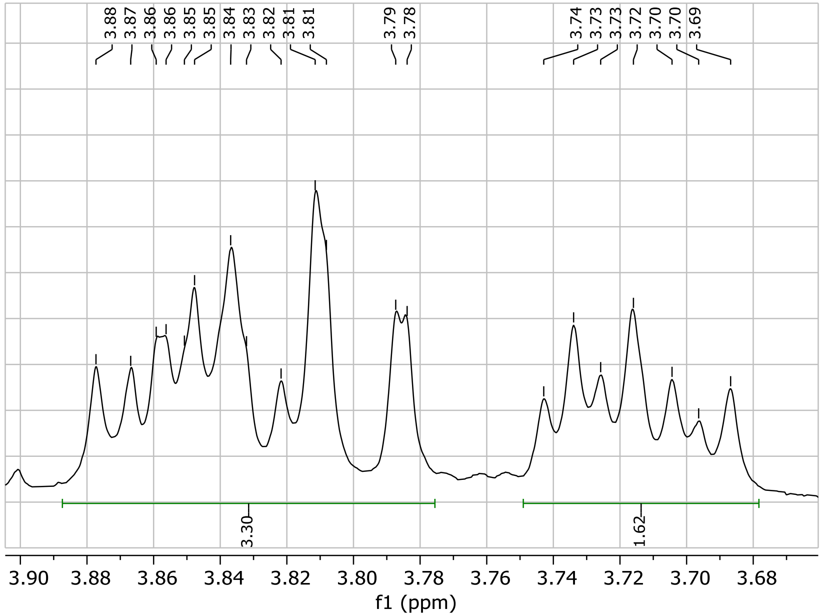
3.88 3.87 3.86 3.86 3.85 3.85 3.84 3.83 3.82 3.81 3.81 3.79 3.78 3.74 3.73 3.73 3.72 3.70 3.70 3.69
3.30 1.62
3.90 3.88 3.86 3.84 3.82 3.80 3.78 3.76 3.74 3.72 3.70 3.68
f1 (ppm)

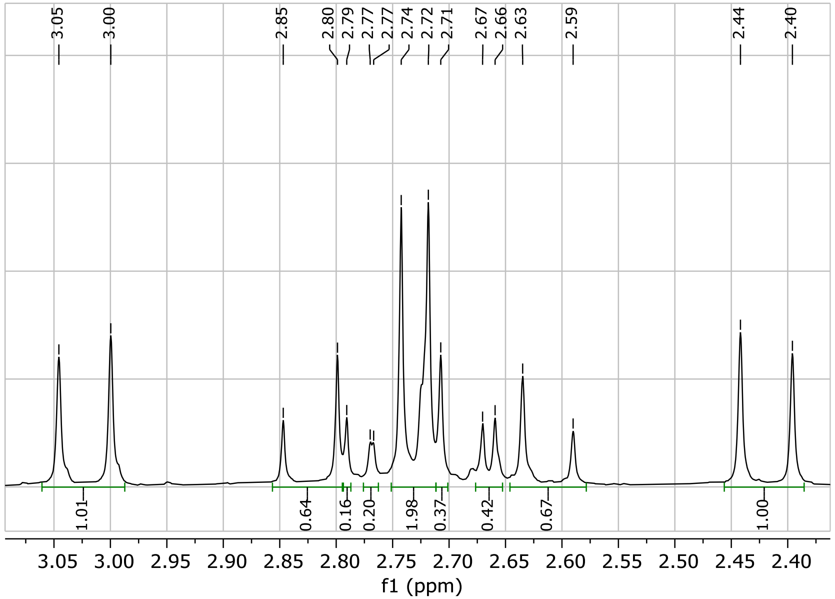
3.05 3.00 2.85 2.80 2.79 2.77 2.77 2.74 2.72 2.71 2.67 2.66 2.63 2.59 2.44 2.40
1.01 0.64 0.16 0.20 1.98 0.37 0.42 0.67 1.00
3.05 3.00 2.95 2.90 2.85 2.80 2.75 2.70 2.65 2.60 2.55 2.50 2.45 2.40
f1 (ppm)

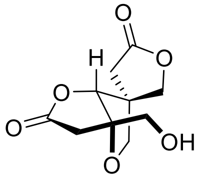

Lappaceolide A (**1**)

+

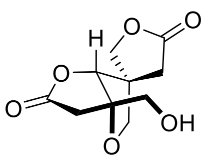

Lappaceolide B (**2**)

$\{^1H\}^{13}C$ NMR, 100 MHz
$CDCl_3$

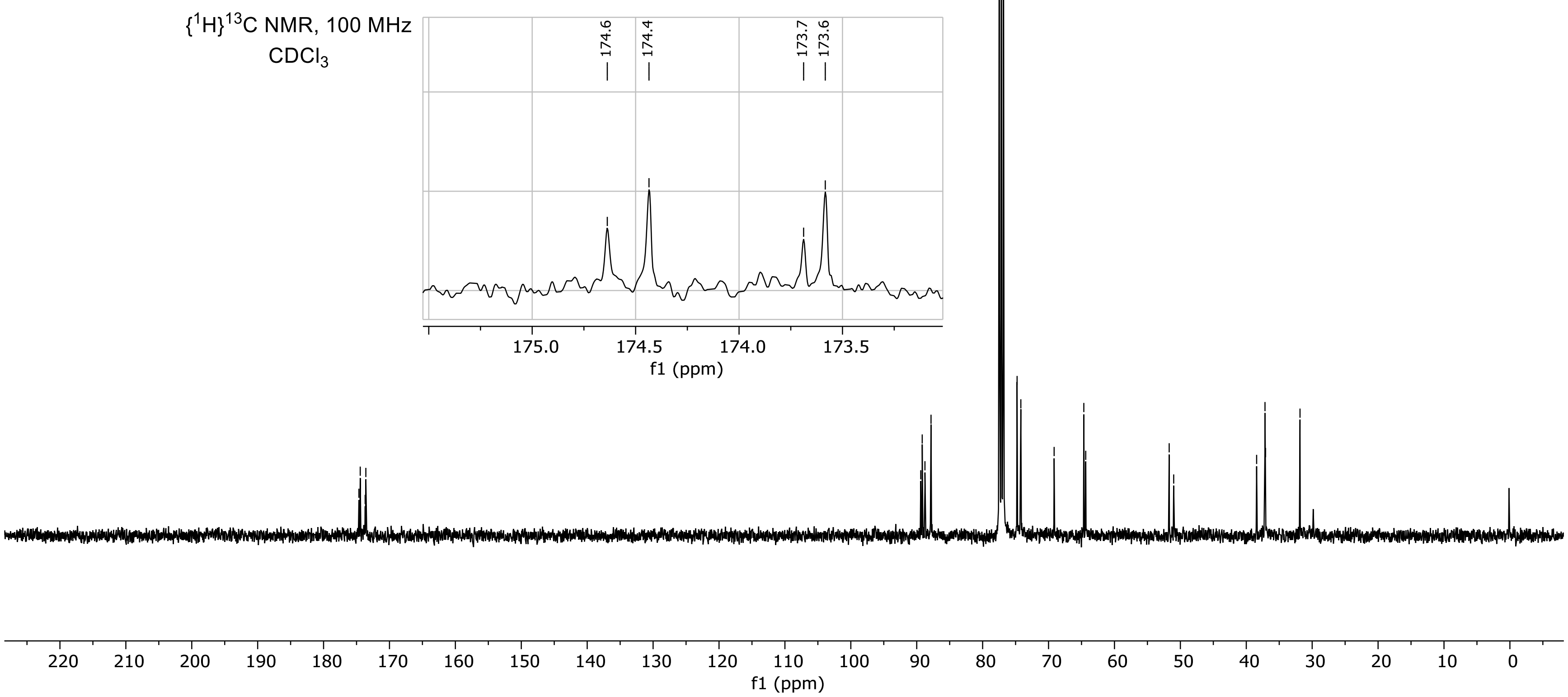

Supplement: Supplementary file 1 [file ol5c02445_si_001.pdf]
